# Faecal calprotectin concentrations in apparently healthy children aged 0-12 years in urban Kampala, Uganda: a community-based survey

**DOI:** 10.1186/1471-2431-11-9

**Published:** 2011-02-02

**Authors:** Elin Hestvik, James K Tumwine, Thorkild Tylleskar, Lena Grahnquist, Grace Ndeezi, Deogratias H Kaddu-Mulindwa, Lage Aksnes, Edda Olafsdottir

**Affiliations:** 1 Centre for International Health, University of Bergen, Årstadveien 21, N-5009 Bergen, Norway; 2 Department of Paediatrics, Haukeland University Hospital, N-5021 Bergen, Norway; 3 Department of Paediatrics and Child Health, Makerere University College of Health Sciences, School of Medicine, P.O Box 7072, Kampala, Uganda; 4 Department of Women's and Children's Health, Karolinska Institutet, 17176 Stockholm, Sweden; 5 Department of Microbiology, Makerere University College of Health Sciences, School of Medicine, School of Biomedical Sciences, P.O Box 7072, Kampala, Uganda; 6 Department of Clinical Medicine, University of Bergen, Bergen, Norway

## Abstract

**Background:**

Calprotectin is a calcium and zinc binding protein, abundant in neutrophils and is extremely stable in faeces. Faecal calprotectin is used as a non-specific marker for gastrointestinal inflammation. It has a good diagnostic precision to distinguish between irritable bowel syndrome and inflammatory bowel disease. Studies have established normal concentrations in healthy children; all these studies have been performed in high-income countries. The objective of this study was to determine the concentration of faecal calprotectin in apparently healthy children aged 0-12 years in urban Kampala, Uganda.

**Method:**

We tested 302 apparently healthy children aged, age 0-12 years (162 female, 140 male) in urban Kampala, Uganda. The children were recruited consecutively by door-to-door visits. Faecal calprotectin was analyzed using a quantitative enzyme-linked immunosorbent assay. Faeces were also tested for *Helicobacter pylori (H. pylori) *antigen, for growth of enteropathogens and microscopy was performed to assess protozoa and helminths. A short standardized interview with socio-demographic information and medical history was obtained to assess health status of the children.

**Results:**

In the different age groups the median faecal calprotectin concentrations were 249 mg/kg in 0 < 1 year (n = 54), 75 mg/kg in 1 < 4 years (n = 89) and 28 mg/kg in 4 < 12 years (n = 159). There was no significant difference in faecal calprotectin concentrations and education of female caretaker, wealth index, gender, habits of using mosquito nets, being colonized with *H. pylori *or having other pathogens in the stool.

**Conclusion:**

Concentrations of faecal calprotectin among healthy children, living in urban Ugandan, a low-income country, are comparable to those in healthy children living in high-income countries. In children older than 4 years, the faecal calprotectin concentration is low. In healthy infants faecal calprotectin is high. The suggested cut-off concentrations in the literature can be used in apparently healthy Ugandan children. This finding also shows that healthy children living under poor circumstances do not have a constant inflammation in the gut. We see an opportunity to use this relatively inexpensive test for further understanding and investigations of gut inflammation in children living in low-income countries.

## Background

Calprotectin is a calcium and zinc binding heterocomplex protein, described by Fagerhol et al. in 1979 [1]. It is abundantly present in the cytosol fraction of neutrophils [2], it is also found in monocytes/macrophages, but is absent from platelets and lymphocytes [3]. It is used as a non-specific marker for activation of granulocytes and mononuclear phagocytes. Calprotectin is remarkably resistant to degradation in the presence of calcium, it is stable in faeces stored for 7 days at room temperature [4] and no changes over time have been found by storing the faeces at -20°C [5]. A faecal calprotectin Enzyme-linked immunosorbent assay (ELISA) test has been available since 1994 [6].

Faecal calprotectin is used as a non-specific marker for gastrointestinal (GI) inflammation. It has been shown to correlate significantly with four day faecal excretion of ^111^indium [7], the gold standard for intestinal inflammation. Faecal calprotectin concentrations are elevated both in adults [4,8] and children [9-11] with inflammatory bowel disease (IBD) and can be used to evaluate the degree of inflammation in these patients. For the diagnosis and more thorough investigation of IBD, colonoscopy is needed. There is a significant correlation between calprotectin concentration in gut lavage fluid and intestinal permeability, suggesting that increased intestinal permeability in IBD might be a consequence of inflammation in the intestinal wall and hereby increased transepithelial migration of neutrophils [12]. Faecal calprotectin may differentiate between irritable bowel disease and IBD in school-age children [13]. Faecal calprotectin is found elevated in adults and children with various GI infections [14-16], but the concentrations are lower than in persons with IBD. Calprotectin is present in plasma, and the faecal calprotectin concentrations might be increased with any bleeding into the GI tract [17]. Elevated concentrations of faecal calprotectin have been described in cystic fibrosis, rheumatoid arthritis, Crohn's disease, ulcerative colitis and bacterial infection [6], as well as neoplastic conditions [17] and Non-Steroidal Anti-Inflammatory Drugs (NSAID) induced enteropathy [18]. In young infants high faecal calprotectin concentrations are normal [10,19]. In healthy pre-term babies the concentrations are comparable with healthy term-babies [20,21], but in very low birth weight babies (VLBW) developing severe abdominal disease for instance necrotizing enterocolitis (NEC), faecal calprotectin concentrations tend to increase even more and it may be a marker for early diagnosis [20,21].

Normal values for faecal calprotectin in different age groups have been investigated in high-income countries [10,20-22]. To our knowledge, there are no published articles on faecal calprotectin concentrations in apparently healthy children living in low-income countries. In order to even discuss the importance of calprotectin in low-income countries, a baseline of healthy children has to be done. A study on faecal calprotectin in Schistosomiasis infected Ugandan children and adults have not shown an increase of faecal calprotectin in the infected persons [23].

The objective of this study was to determine the concentration of faecal calprotectin in apparently healthy children aged 0-12 years in urban Kampala, Uganda.

## Methods

### Study design, site and population

**Figure 1:**
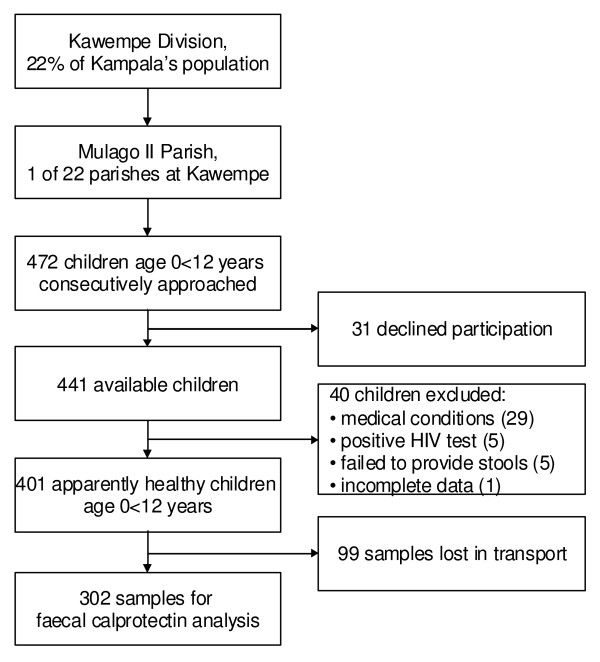
Study profile

This is a cross-sectional survey in apparently healthy children aged 0-12 years in urban Kampala, Uganda. A detailed description is provided elsewhere [24]. Of the 472 children approached, 31 declined participation (6.6%). Forty potential participants (9.1%) were excluded from the final analysis due to positive human immunodeficiency virus (HIV) test (5), incomplete data (1), failed to produce stool within two weeks (5) and medical conditions (29), figure 1. Within the group excluded due to medical condition 23 reported to have ongoing diarrhoea or diarrhoea within last two weeks, two had congenital heart disease, one had a rectal prolapse, two reported to have had nose bleeding within last two weeks and one reported to have peptic ulcer. The youngest child encountered in the survey was one week. An additionally 99 stool samples were lost during transport to the final laboratory. Children reporting chronic cough/asthma were included as no studies have not shown significant elevated concentrations of faecal calprotectin in children with asthma [25].

### Data collection

The data collection took place October-November 2007 in Kampala, Uganda. All stool samples were investigated by microscopy for protozoa and helminths, a culture was performed to assess for enteropathogens and all samples were tested for *Helicobacter pylori *(*H. pylori) *with a rapid faecal monoclonal antigen test [24]. In order to assess the faecal calprotectin concentrations among healthy, non-HIV-infected, children in this high endemic area, all participants and their caretakers were offered a voluntary HIV test.

### Stool sampling and CALPRO^® ^Calprotectin ELISA Test

A stool sample was requested from each participating child. All participants were instructed from the data collectors to pass stool on a newspaper that was handed out and thereafter the stool was collected with the spoon following the air tight containers either at time of the encounter, at the end of the day, or the following morning. All participants who had not delivered a stool sample were visited once daily for the next two weeks. A participant was included in the survey if he/she produced a stool sample within two weeks after the initial interview. Stool samples were transported at ambient temperature from the field to the laboratory twice daily and stored in a +4°C fridge until the same afternoon or the following day when a stool portion was frozen in a clean Eppendorf tube at -80°C. The frozen stool samples were transported on ice by air to Bergen where the final analyses were performed in July 2009 using CALPRO^® ^Calprotectin ELISA Test (ALP). Instructions given by the manufacturer were followed (http://www.calpro.no). Eighty two of 302 faecal calprotectin samples (27%) were measured twice to evaluate the consistency within the pairs. In order to manage the data, all 164 concentrations were ranged into quartiles. The strength of agreement, kappa (95%CI), was very good, 0.81 (0.70-0.92). The CALPRO^® ^Calprotectin ELISA Test (ALP) is a quantitative method for the determination of calprotectin in faeces. Calprotectin was expressed as milligram per kilogram (mg/kg) of faeces. For children younger than 4 years of age there are no reference limits established for a positive test.

### Statistical analysis

The statistical analysis were performed as described elsewhere [24]. The data were exported to SPSS version 17.0 for statistical analysis. The concentration of faecal calprotectin was expected to have a skewed distribution, therefore the median was used. The confidence interval (CI) reported was set to 95%. All tests were 2-sided, p-value of 0.05 or less was considered significant. Faecal calprotectin values in the different groups were compared by using Mann-Whithey U test (for to different groups) and by Kruskal-Wallis H test (for three or more groups). Age was reported in mean and years.

### Ethics

Ethical approval was obtained from Makerere University, Faculty of Medicine, Research and Ethics Committee in Uganda and the Regional Committee for Medical and Health Research Ethics, West-Norway (REK-VEST) in Norway. The data collectors were trained in ethical issues prior to the study. Oral and written information about the study was given to the caretakers either in English or the local language. Informed consent was obtained from all the caretaker of the participants in the study.

## Results

The mean age (±SD) of all the participants was 4.9 (3.6) years, for girls 5.4 (3.7) years and boys 4.4 (3.5) years. For the children above 4 years the mean age (±SD) was 7.9 (2.2), for girls 8.0 (2.3) and boys 7.8 (2.2). Gender was unequally represented in the study, 1) for all participants 162 (53.6%) girls and 140 (46.4%) boys, 2) for the children above 4 years 96 (60.4%) girls and 63 (39.6%) boys.

**Figure 2:**
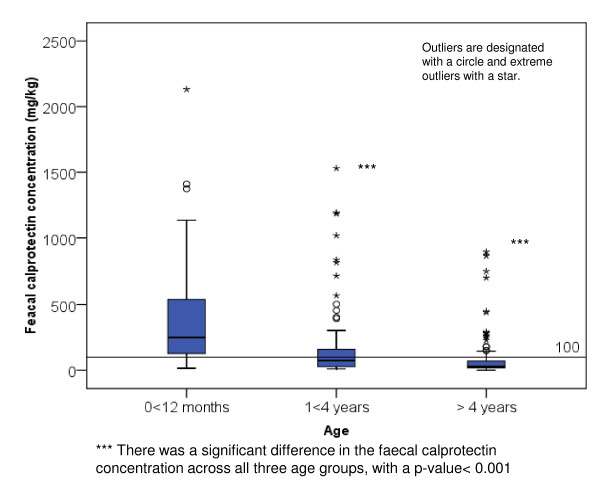
Median faecal calprotectin with 95% CI by age in years

The faecal calprotectin concentration had a skewed distribution in the 302 apparently healthy children, figure 2. In the three age groups the number of children were 54 (0 < 1 year), 89 (1 < 4 years) and 159 (4 < 12 years). The median faecal calprotectin concentrations with 95% CI were 249 mg/kg (180-403) (0 < 1 year), 75 mg/kg (53-119) (1 < 4 years) and 28 mg/kg (25-35) (4 < 12 years), table 1. There was a significant difference in the faecal calprotectin concentrations across all three age groups, regardless of gender. In the younger age group the concentration of faecal calprotectin was more spread and had a lager range than in the older children, where the values were skewed towards the lower end of the scale, figure 2. By dividing the children younger than 1 year into 3 groups, 0 < 3 months (n = 14), 3 < 6 months (n = 13) and 6 < 12 months (n = 27) we found that the youngest children had a trend for highest concentrations of faecal calprotectin (with 95%CI); 354 (195-621) (0 < 3 months), 278 (85-988) (3 < 6 months) and 183 (109-418) (6 < 12 months), but none of this differences were statistically different, table 1.

**Table 1:** Faecal calprotectin concentration in apparently healthy children by age

Age	**Number **(%)	Median FC (mg/kg) (95%CI)
0 < 3 months	14 (4.6)	345 (195-621)
3 < 6 months	13 (4.3)	278 (85-988)
6 < 12 months	27 (8.9)	183 (109-418)
1 < 4 years	89 (29.5)	75 (53-119)*
4 < 12 years	159 (52.6)	28 (25-35) *

All	302 (100)	52 (40-73)

* Difference in median, p-value < 0.001 if compared with the whole group of children younger than 12 months.

CI confidence interval

FC faecal calprotectin

We performed a subgroup analysis of children aged 4 < 12 years where faecal calprotectin has proven to be most useful and where reference values are available. By performing Mann-Whithey U and Kruskal-Wallis H test there was no significant relation between concentration of faecal calprotectin and education of the female caretaker, wealth index, sex, child using a mosquito net regularly, being colonized by *H. pylori, Giardia intestinalis *(*G.intestinalis*) or other pathogens, table 2. Within the last three months before inclusion 28.6% of the children had been treated with antibiotics and 24.2% had been treated for malaria; there were no significant difference in median faecal calprotectin value in the treated versus the not treated participants.

**Table 2:** Faecal Calprotectin concentration in 159 apparently healthy children age 4 < 12 years

	Number N (%)	Median FC concentration (mg/kg) (95% CI)	p-value
**Sex**			
Female	96 (60.4)	27.5 (22-37)	0.94
Male	65 (39.6)	29 (24-38)	
**Education of female caretaker**			
Not completed secondary school	131 (82.4)	28 (24-35)	0.74
Completed secondary	28 (17.6)	28 (20-63)	
**Using a mosquito net**			
Yes	52 (32.7)	28 (21-35)	0.36
No	107 (67.3)	29 (24-40)	
**Wealth index**			
Poor	51 (32.1)	25 (20-34)	0.39
Poorer	55 (34.6)	30 (24-46)	
Poorest	53 (33.3)	29 (22-40)	
***G.intestinalis *seen by microscopy**			
Yes	35 (22.0)	40 (23-34)	0.12
No	124 (78.0)	27.5 (24-88)	
***H. pylori *colonization**			
Yes	78 (49.1)	33 (25-46)	0.12
No	81 (50.9)	26 (22-34)	
**Other pathogens seen by microscopy ª**			
Yes	13 (8.2)	34 (18-46)	0.66
No	146 (91.8)	28 (23-35)	

ª Campylobacter jejuni (1), Hymenolepis nana (5), Entamoeba histolytica (2), Ancylostoma duodenale (3), Ascaris lumbricoides (2)

N number

CI confidence interval

FC faecal calprotectin

Of the 159 children above 4 years, 131 (82.4%) had faecal calprotectin below 100 mg/kg, and 143 of the 159 children (89.9%) older than 4 years, had faecal calprotectin below 150 mg/kg, table 3. Of the 28 children having a faecal calprotectin higher than 100 mg/kg, 11 had an intestinal infection with *G. intestinalis *and 1 had Ancylostoma duodenale. However, in 16 children we did not find any explanation for faecal calprotectin over 100 mg/kg, the mean faecal calprotectin was 295 mg/kg with a maximum of 895 mg/kg. Thirteen of the sixteen were female, had a mean age (±SD) of 7.8 (2.3) years, only three of them were using a mosquito bed net regularly and ten were colonized with *H. pylori*.

**Table 3:** Distribution of the faecal calprotectin in apparently healthy children >4 years

Faecal calprotectin concentration (mg/kg)	Number N	Percent %	Cumulative percent %
<50	111	69.8	69.8
50 < 100	20	12.6	82.4
100 < 150	12	7.5	89.9
≥150	16	10.1	100.0

Total	159	100.0	100.0

N Number

Six participants reported themselves to be chronically ill, five with chronic cough/asthma and one with headache, all of them had a faecal calprotectin less than 40 mg/kg. In one culture only an enteric pathogen was isolated; Campylobacter spp. The child was 10 years old and the faecal calprotectin was 43 mg/kg.

The mean age (±SD) in the children whom stool was lost in transport was 4.5 (3.7) years, with more boys (58.6%) than girls (41.4%). Colonization rate with *H. pylori *was 36.4%. The education of the female caretaker, the practice of using mosquito net and the wealth index were similar to the once completed the survey.

## Discussion

This is the first survey of faecal calprotectin concentrations in an apparently healthy population in Sub-Saharan Africa. We have shown that cut-off values recommended to use in children in high-income countries living in a relatively "clean environment" also are valid in children in a low-income country. In our study the median faecal calprotectin in apparently healthy children older than 4 years was 28 mg/kg and within the suggested cut-off concentrations for the test used. By comparing our findings to other studies looking at apparently healthy children, our median with 95% CI is comparable to those studies [9,10,16,22,26,27].

Since none of the children with elevated faecal calprotectin concentrations were followed up to see if the concentrations normalized over time, we do not have an explanation for faecal calprotectin higher than 100 mg/kg in 16 children. Spontaneous normalization in faecal calprotectin concentration without disease has been described [22]. Use of NSAID is one common explanation we did not control for [18]. The participants did not go through a clinical examination and anal fissures with bleeding or colon polyps as described in other studies [28], could contribute to the increased concentration of faecal calprotectin. We excluded all children reporting diarrhoea within last two weeks before encountered in the survey, but some few children could be carrier of asymptomatic intestinal infection from pathogens we have not examined for, for instance Cryptosporidia, Some children with protozoa or helminths might have been missed due to single sample investigation and without additional tests. Ideally, identification of protozoa and helminths are done using 3 consecutive stool samples [29,30]. A strength of our survey is that our children were clinically healthy without diarrhoea and were HIV negative. Another strength is that only one stool culture was positive. Our study was preformed with the improved faecal calprotectin assay, and it has been argued that it gives a better separation between normal and pathologic values [5]. We also adjusted for age within the group of children age 4 < 12 years by applying bivariable linear regression (not shown), but we did not find any changes.

There are few studies on faecal calprotectin and GI-infections [15]. Colonization with *G. intestinalis *and *H. pylori *are common in children living in Sub-Saharan Africa [24,31,32]. In this survey we have found comparable colonization rates. Despite this the median faecal calprotectin was within the recommended cut-off; 37.5 mg/kg for *G. intestinalis *and 33 mg/kg for *H. pylori*. The findings in the *G. intestinalis *infected group were comparable to a Norwegian study in adults with chronic abdominal pain after *G. intestinalis *infection [14], where they found a median faecal calprotectin concentration of 28 mg/kg in the *G. intestinalis *positive patients. Colonization with *H. pylori *can cause changes in the gastric mucosa [33], but there are no reports of increased inflammation in the lower GI tract. Upper-GI disorders have showed little increase in faecal calprotectin levels [34]. Tibble et al. 2002 [35] found faecal calprotectin above the cut-off limit in participants infected by *G. intestinalis *in their study, but those were symptomatic with diarrhoea.

Low-and middle-income countries are reporting an increase in the incidence of IBD since the 1990'ties [36]. To the best of our knowledge there are no studies on the prevalence of IBD in black children living in Sub-Saharan Africa. In addition children living in low-income countries have a higher burden of GI diseases including the effect of HIV on the GI tract. This brings up the need for good methods for improved diagnostics and awareness of GI disorders. This study shows that apparently healthy children do not have an ongoing inflammatory process in the GI tract, and that methods used in high-income countries with a lower burden of GI infection disease also are valid in low-and middle-income countries. There is an ongoing discussion on which upper limit, 100 mg/kg versus 50 mg/kg, provides the best accuracy in diagnosing IBD [37]. For the test we used, an upper limit of 50 mg/kg has been suggested [5,38]. Tibble et al. 2000 used a cut-off concentration of 30 mg/l [7], which is equal to 150 mg/kg [5]. If we use a cut-off of 100 mg/kg, 82.4% of the children had concentrations below, if we use 150 mg/kg, 89.9% of the children had faecal calprotectin concentrations within that range, table 3. A recently published meta-analysis concludes that faecal calprotectin gives a diagnostic precision in distinguishing IBD from non-IBD diagnosis, with higher precision at a cut-off of 100 mg/kg versus 50 mg/kg [37].

Fagerberg et al [22] have documented that the same cut-off limits used in adults are also applicable in children older than 4 years. In infants and toddlers there are no recommended cut-off values. In our study they had higher faecal calprotectin concentrations than children older than 4 years. The concentrations were comparable to those found in other studies of apparently healthy children [10,27]. Our findings contribute to establish reference values also for children younger than 4 years of age. We did not look at feeding practice in children younger than 1 year. Studies diverge in the conclusions if faecal calprotectin is higher in exclusively breast feed children than in mix feed children [39,40].

There were no differences in median faecal calprotectin according to sex [9,10,16], wealth index, health behaviour or education level of female caretaker. This is to our knowledge demonstrated for the first time.

## Conclusion

Apparently healthy Ugandan children, age 4 < 12 years, have comparable concentrations of faecal calprotectin to similar aged children in high-income countries. The concentration of faecal calprotectin is high in Ugandan children under 1 year of age, and is raised in toddlers. Faecal calprotectin can be used in combination with extended history and stool microscopy as a diagnostic tool in children in need for further investigation for prolonged diarrhoea in a limited recourse setting. Faecal calprotectin concentrations over 100 mg/kg in children warrant follow-up. We see an opportunity to use this relatively inexpensive test for further understanding and investigations of gut inflammation in children living in low-income countries.

## Competing interests

The authors declare that they have no competing interests.

## Authors' contributions

EH participated in the conception, design and implementation of the study, statistical analysis, interpretation and writing the manuscript. JKT participated in conception, design and implementation of the study. TT participated in the conception and design of the study, statistical analysis, interpretation and writing the manuscript. LG participated in design of the study, interpretation and writing the manuscript. GN participated in design and implementation of the study. DKM participated in implementation of the study and preparation of the stool for calprotectin analysis. LA participated in performing the faecal calprotectin analysis, statistical analysis, interpretation and writing the manuscript. EO participated in the conception and design of the study, statistical analysis, interpretation and writing the manuscript. All authors read and approved the final manuscript.

## Pre-publication history

The pre-publication history for this paper can be accessed here:

http://www.biomedcentral.com/1471-2431/11/9/prepub
